# Supplementary material for: Evaluation of Direct Detection Protocols for Poliovirus from Stool Samples of Acute Flaccid Paralysis Patients
Source: Viruses. 2023 Oct 18;15(10):2113. doi: 10.3390/v15102113 (PMC10612058; doi:10.3390/v15102113)
Supplement: Supplementary file 1 [file viruses-15-02113-s001.zip › viruses-2652360-supplementary.pdf]

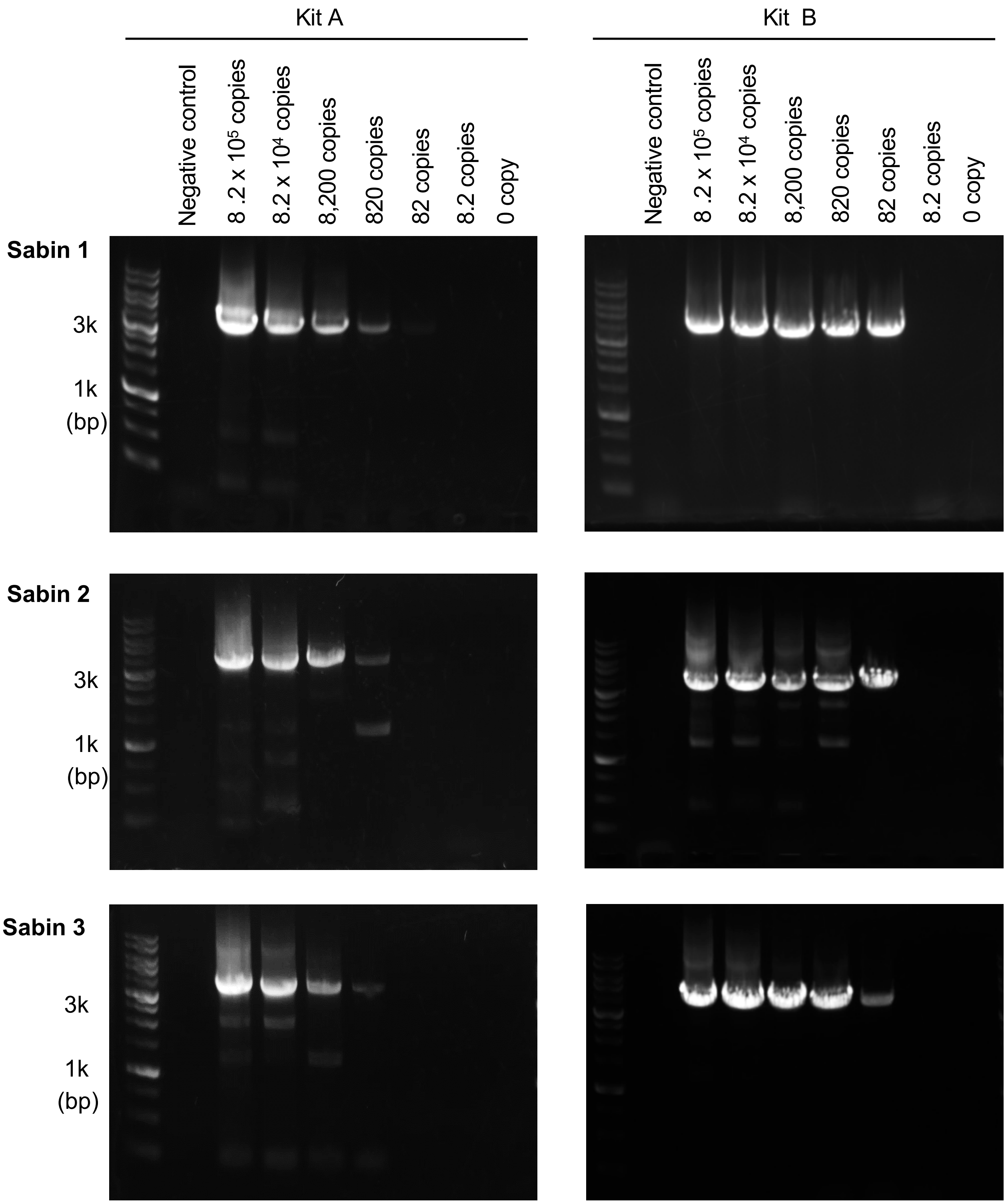

LOD of Sabin 1, 2 and 3: EC RT-PCR (3.9k bp)

Fig.S1

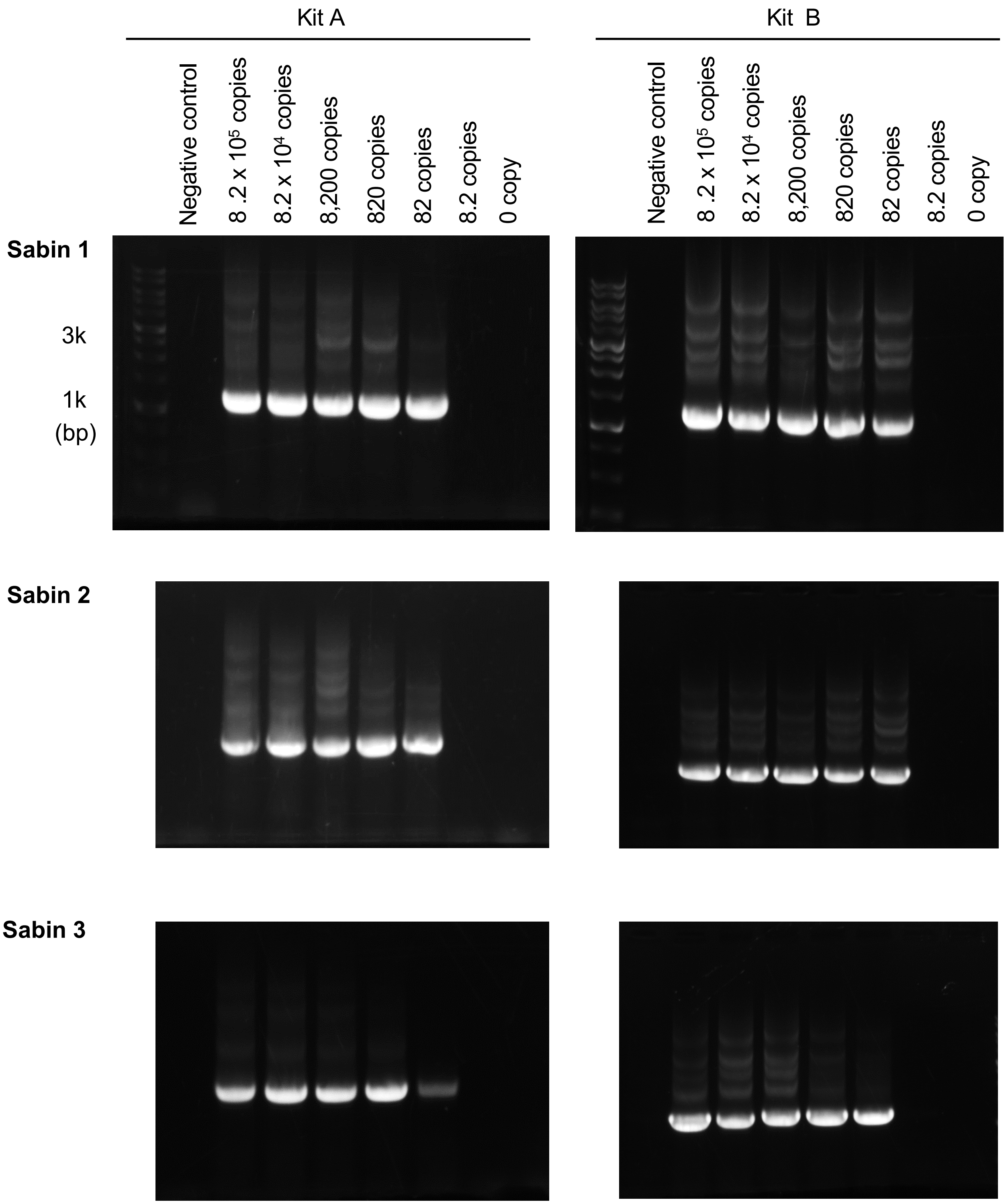

LOD of Sabin 1, 2 and 3 : Pan-PV-VP1 PCR with barcoded primers (1.2k bp)

Fig.S2

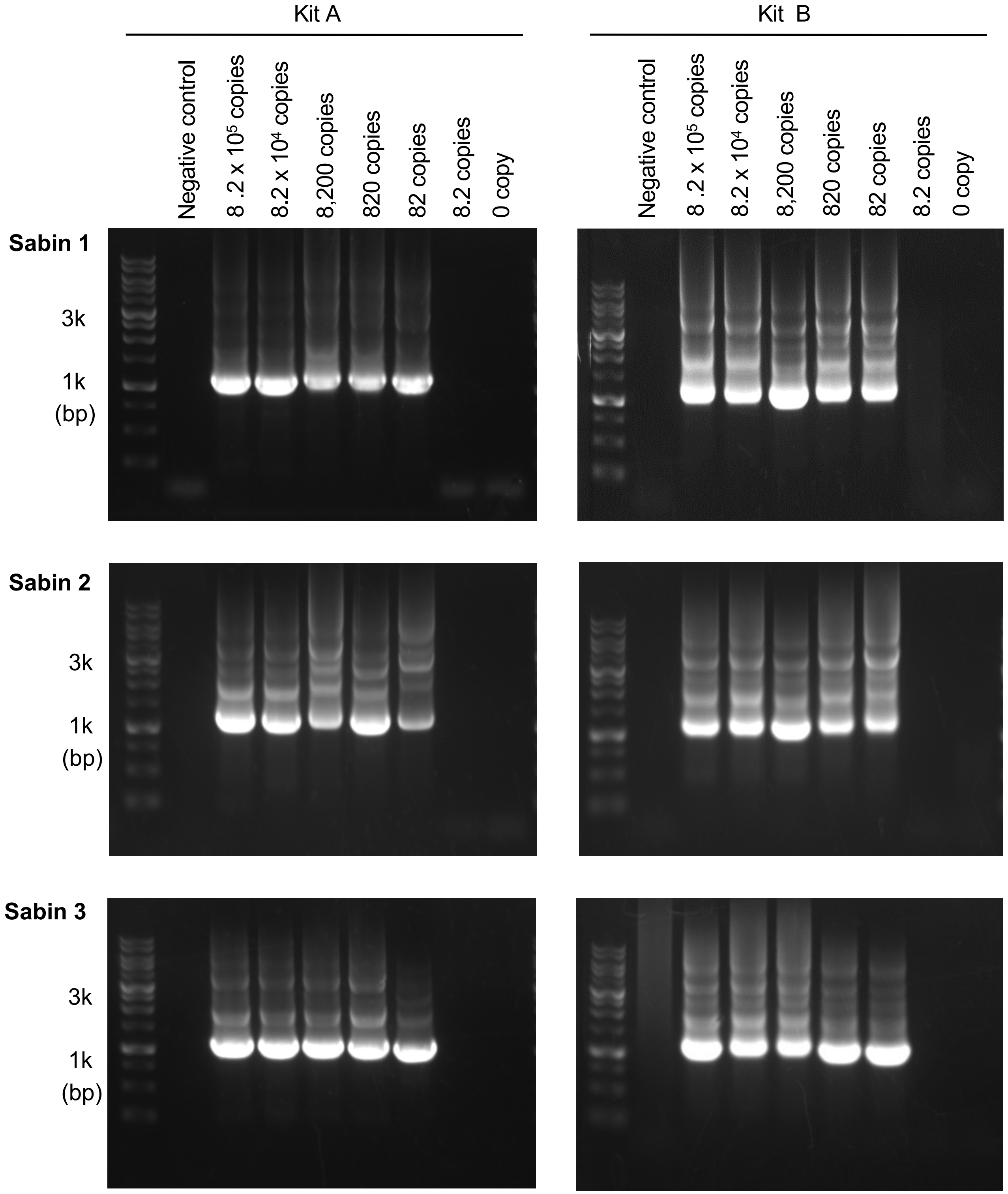

LOD of Sabin 1, 2 and 3 : Pan-PV-VP1 PCR (1.0k bp)

Fig.S3

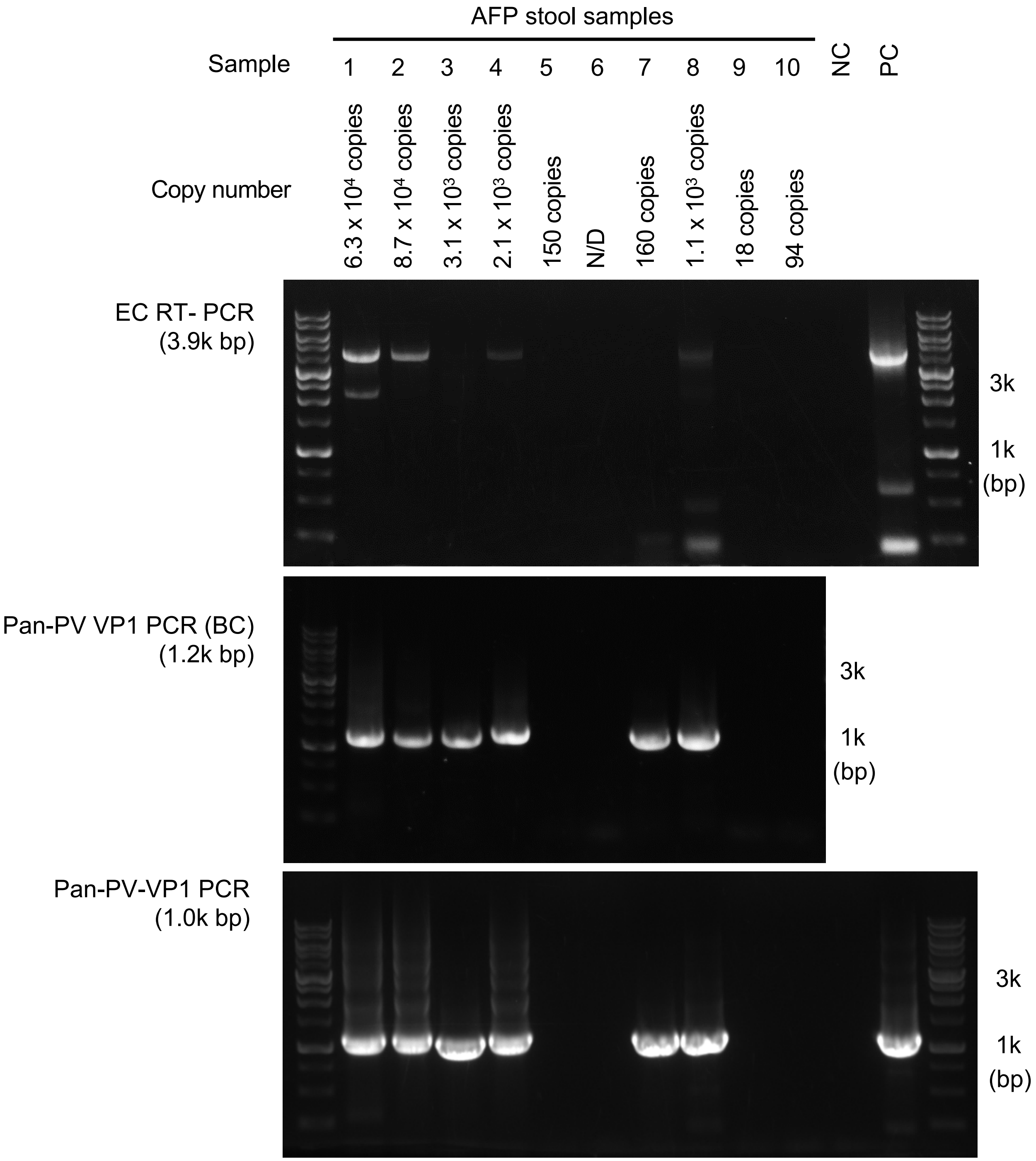

DD of PV from stool samples of AFP cases by kit A: Detection of VP1 region _1

Fig.S4

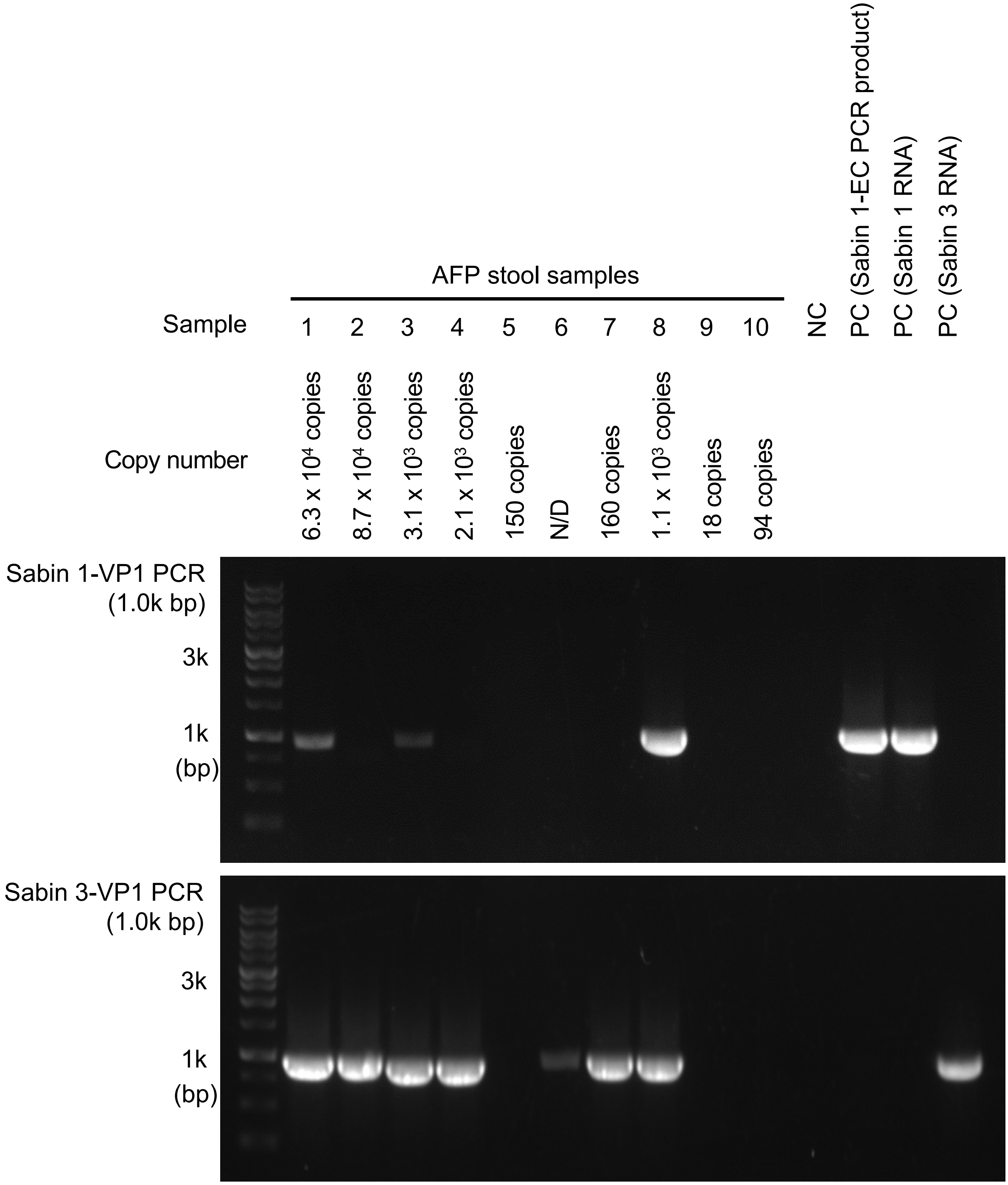

DD of PV from stool samples of AFP cases by kit A: Detection of VP1 region _2

Fig.S5

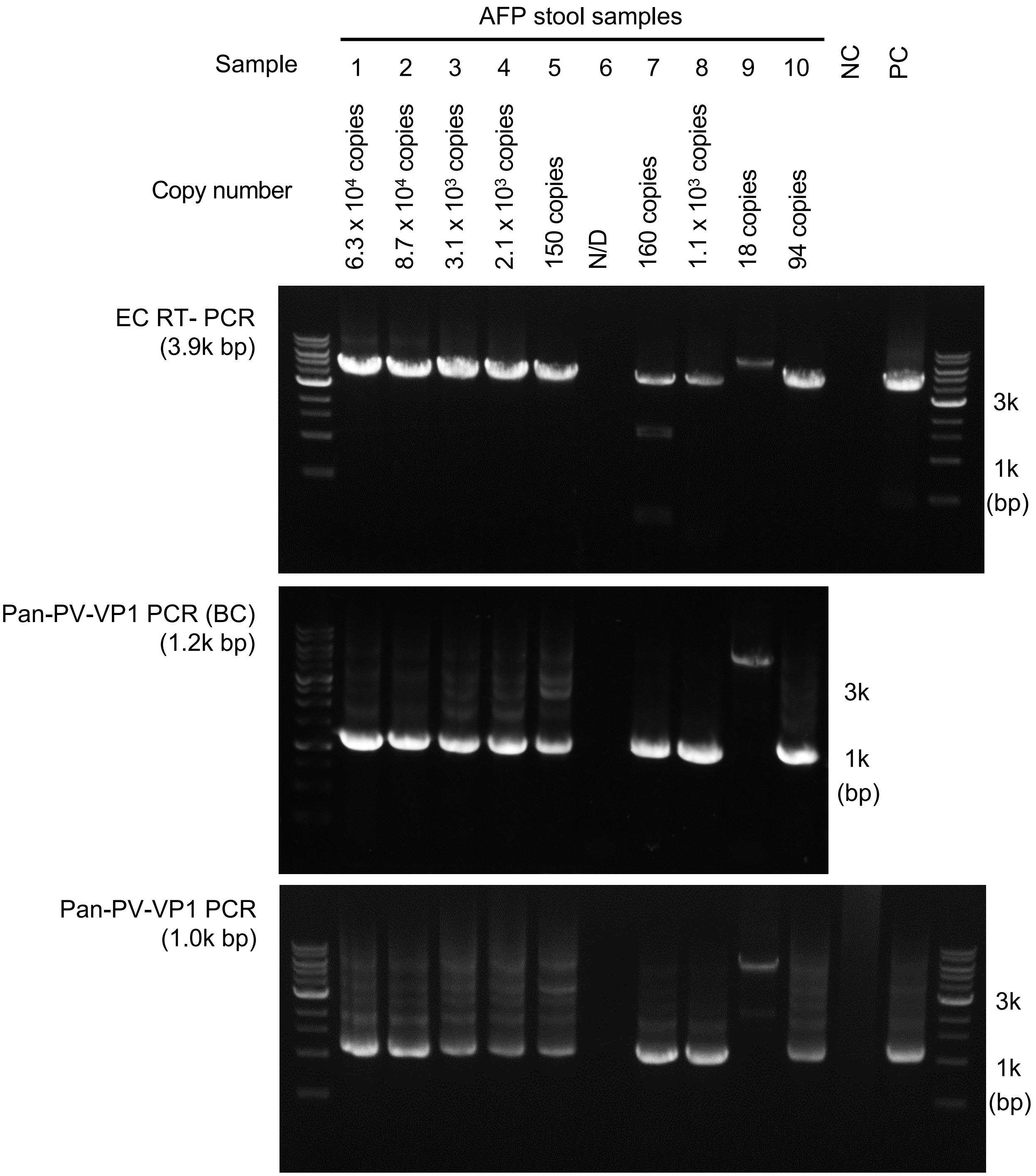

DD of PV from stool samples of AFP cases by kit B: Detection of VP1 region _1

Fig.S6

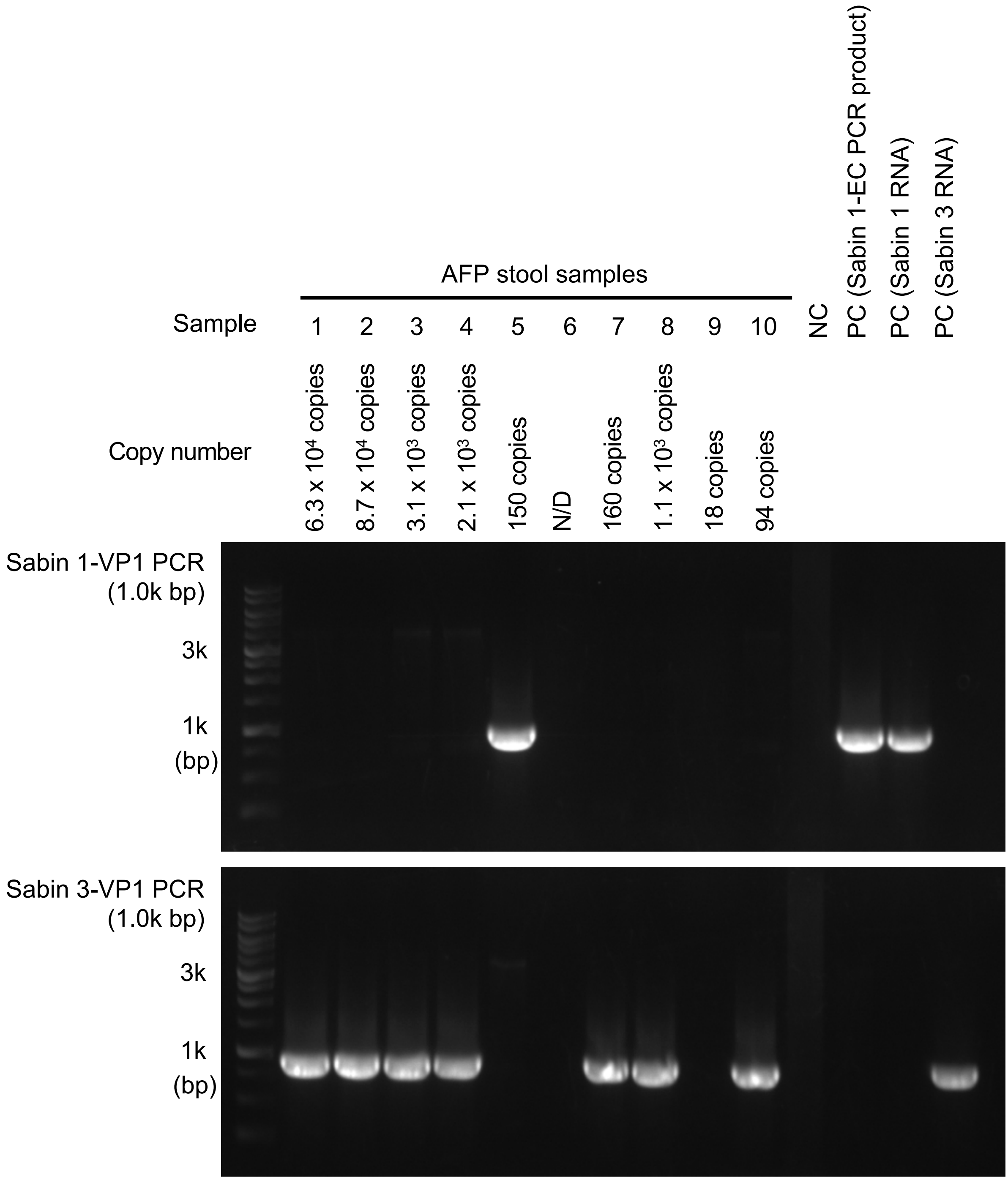

DD of PV from stool samples of AFP cases by kit B: Detection of VP1 region _2

Fig.S7

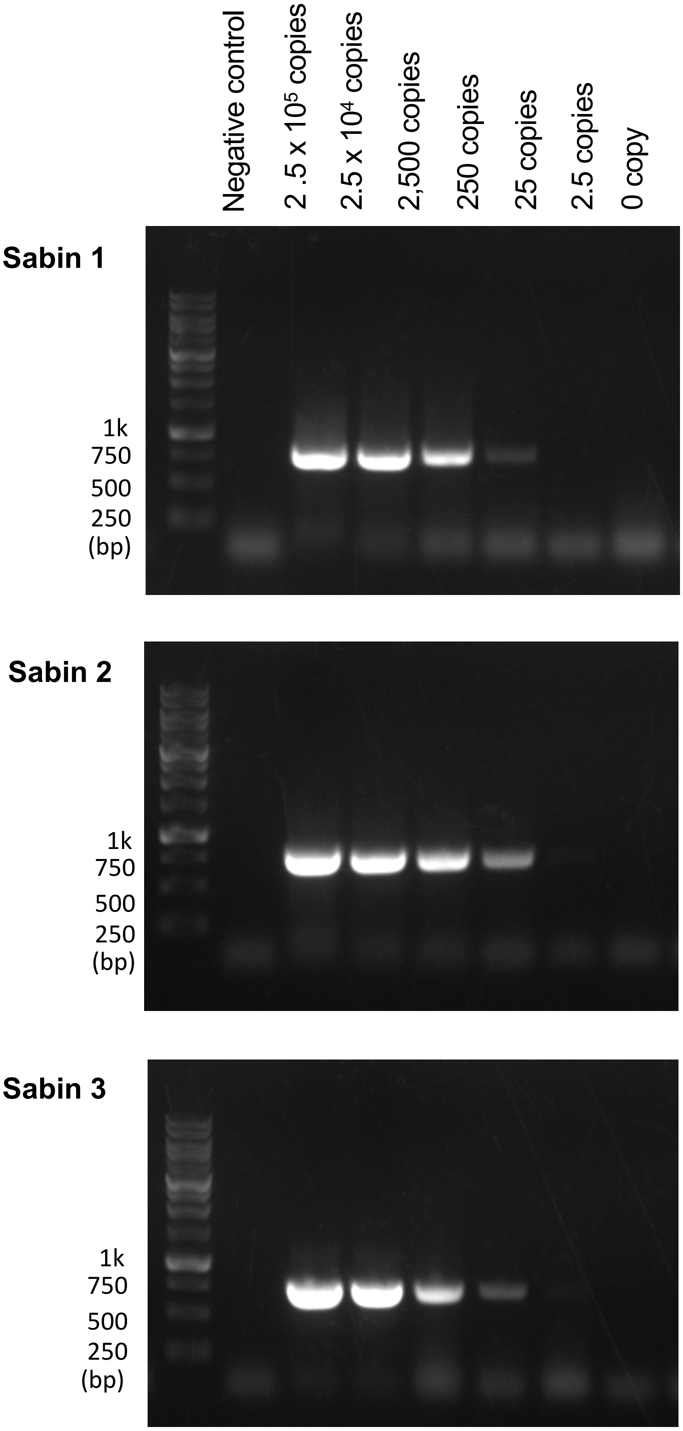

LOD of Sabin 1, 2 and 3: VP4-VP2 semi-nested RT-PCR (1st PCR: 750 bp)

Fig.S8

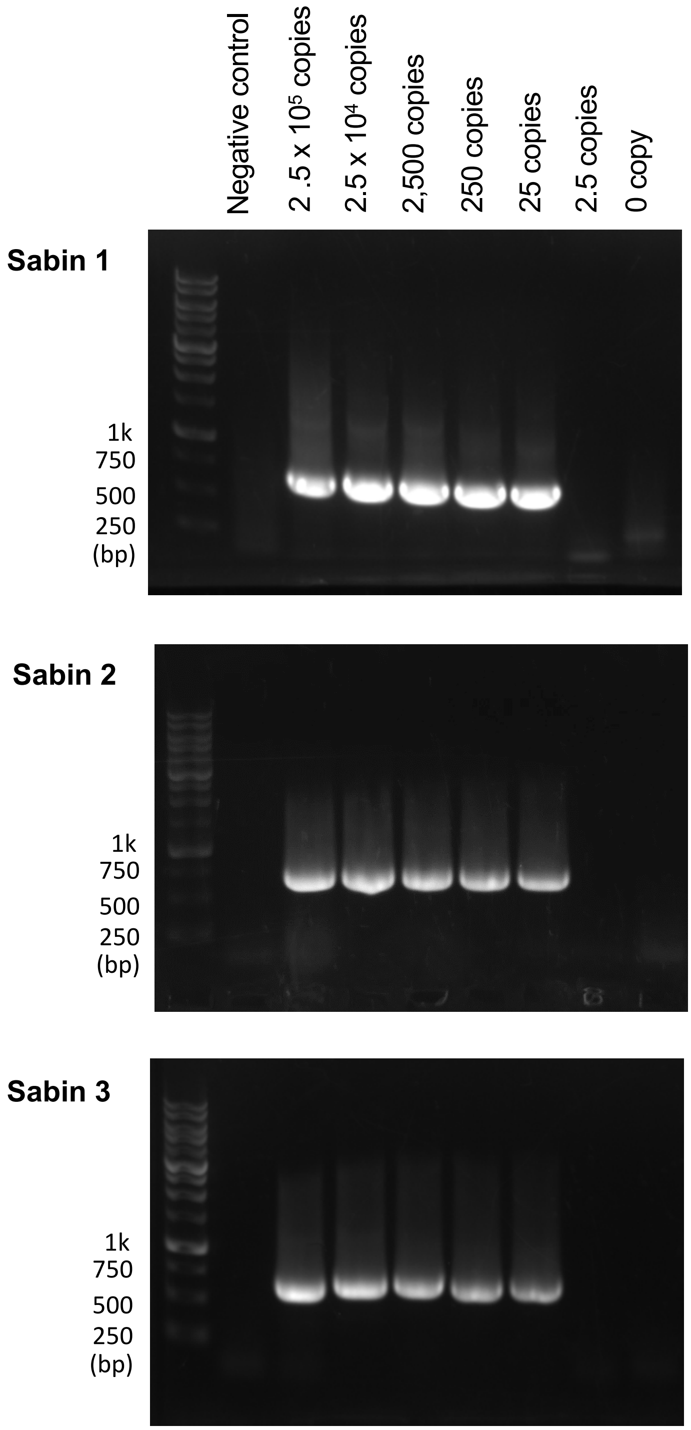

LOD of Sabin 1, 2 and 3: VP4-VP2 semi-nested RT-PCR (2nd PCR: 650 bp)

Fig.S9

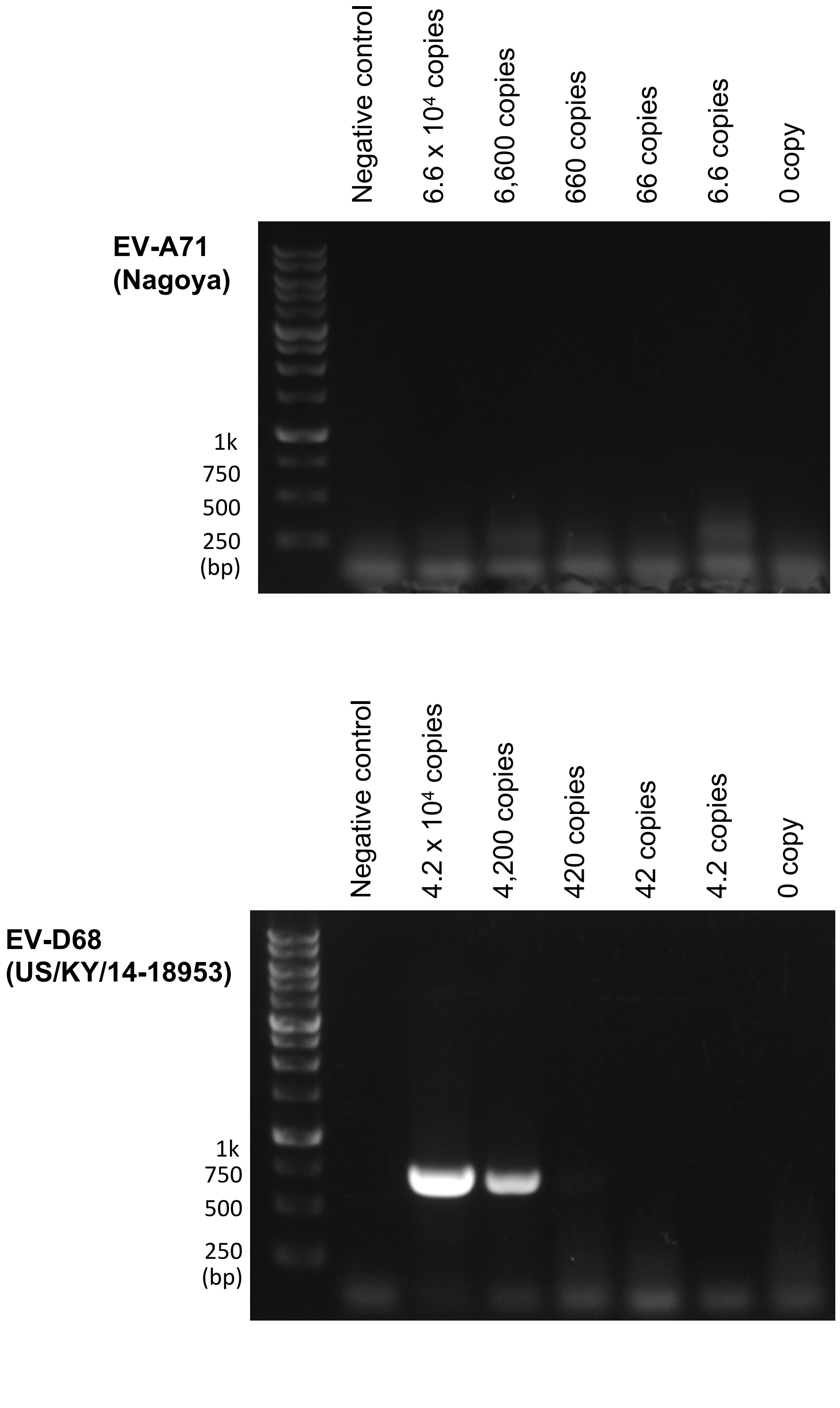

LOD of EV-A71 (Nagoya) and EV-D68 (US/KY/14-18953)
: VP4-VP2 semi-nested RT-PCR (1st PCR: 750 bp)

Fig.S10

EV-A71 (Nagoya)

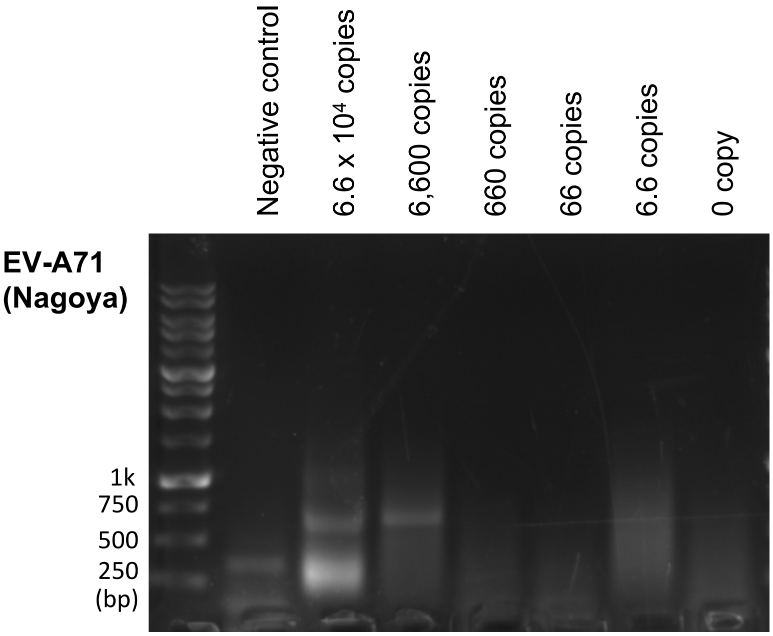

EV-D68 (US/KY/14-18953)

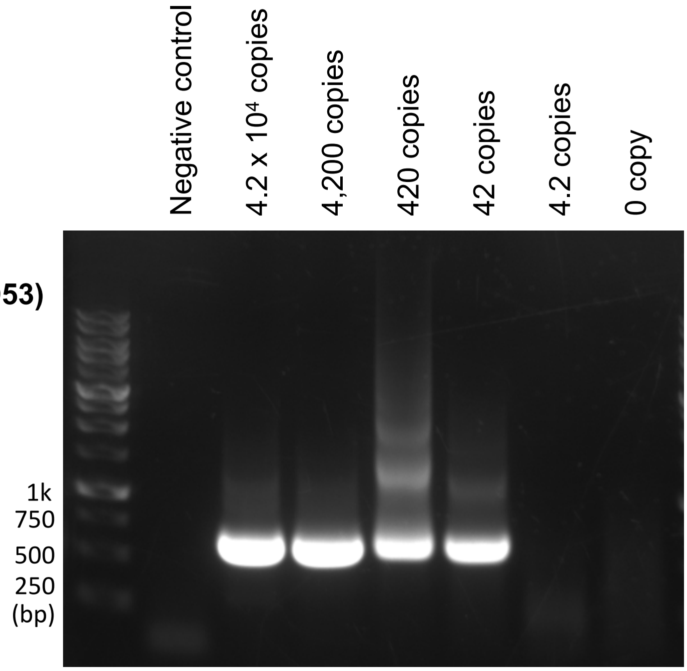

LOD of EV-A71 (Nagoya) and EV-D68 (US/KY/14-18953)
: VP4-VP2 semi-nested RT-PCR (2nd PCR: 650 bp)

Fig.S11

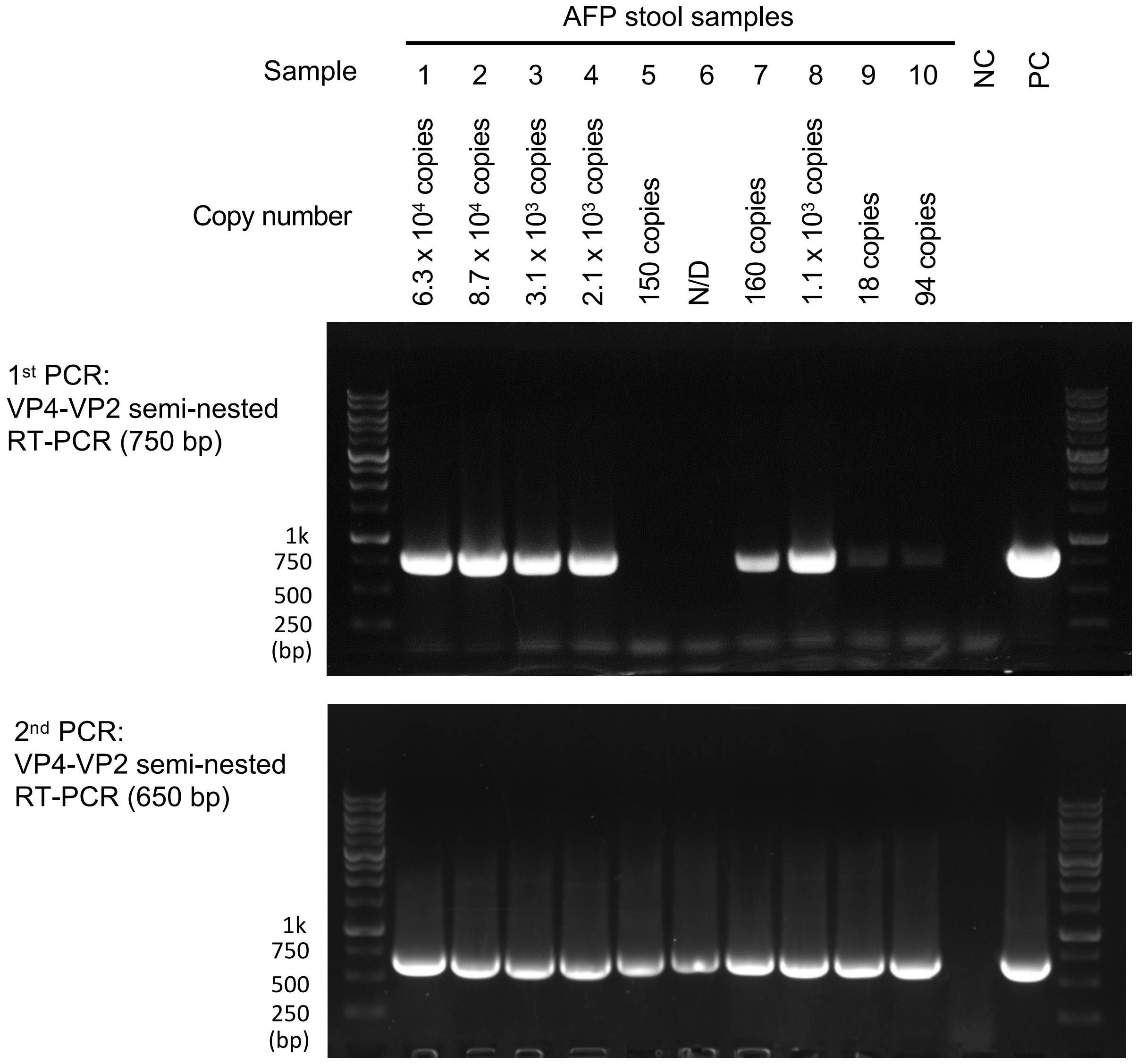

DD of PV from stool samples of AFP cases: Detection of VP4-VP2 region

Fig.S12
